# 2-(4-Chloro­phen­yl)-2-oxoethyl naphthalene-1-carboxyl­ate

**DOI:** 10.1107/S1600536813006958

**Published:** 2013-03-16

**Authors:** B. Garudachari, Arun M. Isloor, Thomas Gerber, Eric Hosten, Richard Betz

**Affiliations:** a National Institute of Technology-Karnataka, Department of Chemistry, Surathkal, Mangalore 575 025, India; b Nelson Mandela Metropolitan University, Summerstrand Campus, Department of Chemistry, University Way, Summerstrand, PO Box 77000, Port Elizabeth, 6031, South Africa

## Abstract

In the title compound, C_19_H_13_ClO_3_, an ester of 1-naphthoic acid with an aromatic alcohol, the least-squares planes defined by the C atoms of the respective aromatic systems enclose an angle of 77.16 (3)°. In the crystal, C—H⋯O contacts connect the mol­ecules into undulating sheets parallel to the *bc* plane.

## Related literature

For general information about phenyl benzoates, see: Rather & Reid (1919 ▶). For the photolytic properties of phenyl benzoates, see: Sheehan & Umezaw (1973 ▶); Ruzicka *et al.* (2002 ▶); Litera *et al.* (2006 ▶). For synthetic applications of phenyl benzoates, see: Huang *et al.* (1996 ▶); Gandhi *et al.* (1995 ▶). For graph-set analysis of hydrogen bonds, see: Etter *et al.* (1990 ▶); Bernstein *et al.* (1995 ▶).
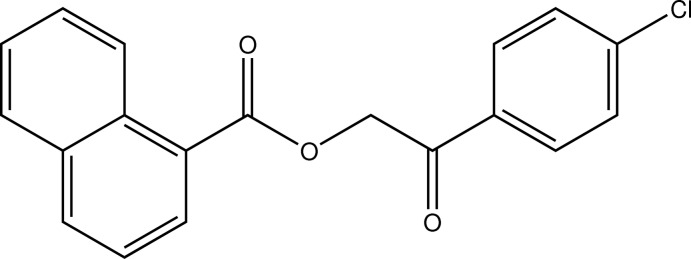


## Experimental

### 

#### Crystal data

C_19_H_13_ClO_3_
*M* *_r_* = 324.74
Monoclinic, 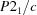

*a* = 5.2708 (1) Å
*b* = 14.8465 (4) Å
*c* = 19.8427 (5) Å
β = 100.383 (1)°
*V* = 1527.32 (6) Å^3^
*Z* = 4
Mo *K*α radiation
μ = 0.26 mm^−1^
*T* = 200 K
0.37 × 0.35 × 0.27 mm

#### Data collection

Bruker APEXII CCD diffractometer
Absorption correction: multi-scan (*SADABS*; Bruker, 2008 ▶) *T*_min_ = 0.941, *T*_max_ = 1.000
14592 measured reflections
3769 independent reflections
2984 reflections with *I* > 2σ(*I*)
*R*_int_ = 0.016

#### Refinement

*R*[*F* ^2^ > 2σ(*F* ^2^)] = 0.035
*wR*(*F* ^2^) = 0.103
*S* = 1.02
3769 reflections
208 parameters
H-atom parameters constrained
Δρ_max_ = 0.30 e Å^−3^
Δρ_min_ = −0.35 e Å^−3^

### 

Data collection: *APEX2* (Bruker, 2010 ▶); cell refinement: *SAINT* (Bruker, 2010 ▶); data reduction: *SAINT*; program(s) used to solve structure: *SHELXS97* (Sheldrick, 2008 ▶); program(s) used to refine structure: *SHELXL97* (Sheldrick, 2008 ▶); molecular graphics: *ORTEP-3 for Windows* (Farrugia, 2012 ▶) and *Mercury* (Macrae *et al.*, 2008 ▶); software used to prepare material for publication: *SHELXL97* and *PLATON* (Spek, 2009 ▶).

## Supplementary Material

Crystal structure: contains datablock(s) I, global. DOI: 10.1107/S1600536813006958/kq2003sup1.cif

Structure factors: contains datablock(s) I. DOI: 10.1107/S1600536813006958/kq2003Isup2.hkl

Supplementary material file. DOI: 10.1107/S1600536813006958/kq2003Isup3.cdx

Supplementary material file. DOI: 10.1107/S1600536813006958/kq2003Isup4.cml

Additional supplementary materials:  crystallographic information; 3D view; checkCIF report

## Figures and Tables

**Table 1:** Hydrogen-bond geometry (Å, °)

*D*—H⋯*A*	*D*—H	H⋯*A*	*D*⋯*A*	*D*—H⋯*A*
C16—H16⋯O1^i^	0.95	2.41	3.2583 (16)	148
C23—H23⋯O3^ii^	0.95	2.48	3.2618 (19)	140

Symmetry codes: (i) 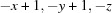; (ii) 
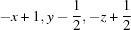
.

**Fig. 1.**
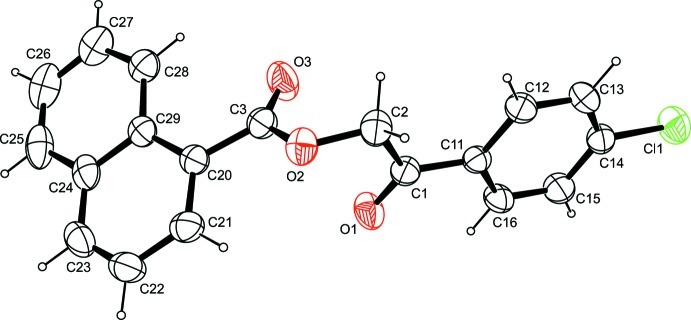
The molecular structure of the title compound, with atom labels and anisotropic displacement ellipsoids (drawn at 50% probability level).

**Fig. 2.**
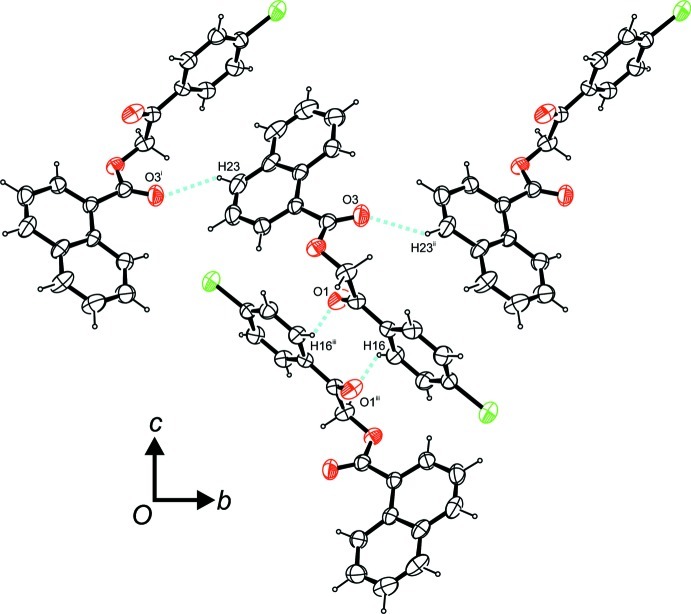
Intermolecular contacts, viewed along [-1 0 0]. Symmetry operators: i -x + 1, y - 1/2, -z + 1/2; ii -x + 1, y + 1/2, -z + 1/2; iii -x + 1, -y + 1, -z.

**Fig. 3.**
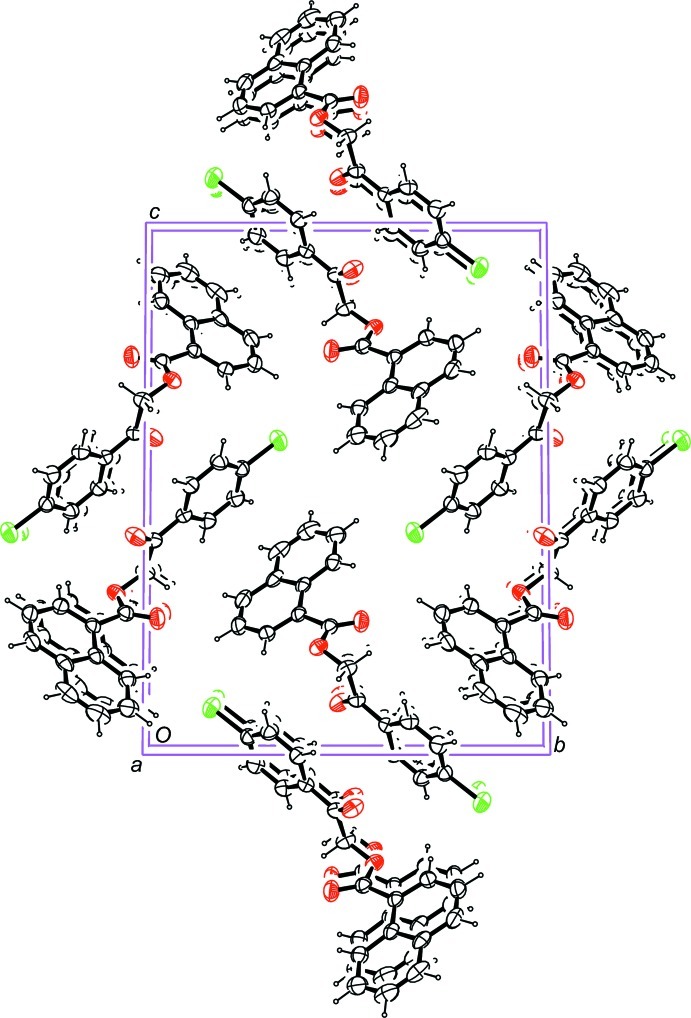
Molecular packing of the title compound, viewed along [-1 0 0] (anisotropic displacement ellipsoids drawn at 50% probability level).

## supplementary crystallographic information

### Comment

For decades, phenyl benzoate derivatives have found ample application in the identification of organic acids (Rather & Reid 1919). These compounds can be photolysed under neutral and mild conditions (Sheehan & Umezaw, 1973; Ruzicka *et al.*, 2002; Litera *et al.*, 2006). They also find application in the field of synthetic chemistry such as in the synthesis of oxazoles and imidazoles (Huang *et al.*, 1996) as well as benzoxazepine (Gandhi *et al.*, 1995). In continuation of our research focused on the crystal structures of medical compounds, the title compound was synthesized.

The planes defined by the atoms of the carboxy group on the one hand and the non-hydrogen atoms of the CH_2_–C═O moiety intersect at an angle of 77.9 (2) °. The least-squares planes defined by the carbon atoms of the respective aromatic systems enclose an angle of 77.16 (3) ° (Fig. 1).

In the crystal, intermolecular C–H···O contacts are observed whose range falls by more than 0.2 Å below the sum of van-der-Waals radii of the atoms participating. One of the hydrogen atoms of the chlorinated phenyl moiety and the oxygen atom of the keto group give rise to centrosymmetric dimers. Additionally, the hydrogen atom in *para* position to the carboxy moiety forms a C–H···O contact to the double bonded oxygen atom of exactly this group in a neighbouring molecule. In total, the molecules are connected to undulated sheets parallel *bc*. In terms of graph-set analysis (Etter *et al.*, 1990; Bernstein *et al.*, 1995), the descriptor for these contacts is *C*^1^_1_(7)*R*^2^_2_(10) on the unary level. Information about metrical parameters as well as the symmetry of those contacts has been summarized in Table 1. The shortest intercentroid distance between two aromatic systems was measured at 4.7603 (9) Å and is apparent between the two different rings in the naphthoic acid moiety and its symmetry-generated equivalents (Fig. 2).

The packing of the title compound in the crystal structure is shown in Figure 3.

### Experimental

A mixture of naphthalene-1-carboxylic acid (0.1 g, 0.0005 mol), potassium carbonate (0.087 g, 0.00063 mol) and 2-bromo-1-(4-chlorophenyl)ethanone (0.147 g, 0.00063 mol) in dimethylformamide (5 ml) was stirred at room temperature for 2 h. After completion of the reaction, the reaction mixture was poured into ice-cold water. The solid product obtained was filtered, washed with water and recrystallized from ethanol (yield: 0.180 g, 95.7%).

### Refinement

Carbon-bound H atoms were placed in calculated positions (C–H 0.95 Å for aromatic carbon atoms and C–H 0.99 Å for methylene groups) and were included in the refinement in the riding model approximation, with *U*(H) set to 1.2*U*_eq_(C).

### Crystal data

**Table:**

C_19_H_13_ClO_3_	*F*(000) = 672
*M**_r_* = 324.74	*D*_x_ = 1.412 Mg m^−^^3^
Monoclinic, *P*2_1_/*c*	Melting point = 396–394 K
Hall symbol: -P 2ybc	Mo *K*α radiation, λ = 0.71073 Å
*a* = 5.2708 (1) Å	Cell parameters from 6659 reflections
*b* = 14.8465 (4) Å	θ = 2.5–28.3°
*c* = 19.8427 (5) Å	µ = 0.26 mm^−^^1^
β = 100.383 (1)°	*T* = 200 K
*V* = 1527.32 (6) Å^3^	Block, colourless
*Z* = 4	0.37 × 0.35 × 0.27 mm

### Data collection

**Table:**

Bruker APEXII CCD diffractometer	3769 independent reflections
Radiation source: fine-focus sealed tube	2984 reflections with *I* > 2σ(*I*)
Graphite monochromator	*R*_int_ = 0.016
φ and ω scans	θ_max_ = 28.3°, θ_min_ = 2.1°
Absorption correction: multi-scan (*SADABS*; Bruker, 2008)	*h* = −7→4
*T*_min_ = 0.941, *T*_max_ = 1.000	*k* = −19→19
14592 measured reflections	*l* = −26→26

### Refinement

**Table:**

Refinement on *F*^2^	Primary atom site location: structure-invariant direct methods
Least-squares matrix: full	Secondary atom site location: difference Fourier map
*R*[*F*^2^ > 2σ(*F*^2^)] = 0.035	Hydrogen site location: inferred from neighbouring sites
*wR*(*F*^2^) = 0.103	H-atom parameters constrained
*S* = 1.02	*w* = 1/[σ^2^(*F*_o_^2^) + (0.0498*P*)^2^ + 0.4268*P*] where *P* = (*F*_o_^2^ + 2*F*_c_^2^)/3
3769 reflections	(Δ/σ)_max_ = 0.001
208 parameters	Δρ_max_ = 0.30 e Å^−^^3^
0 restraints	Δρ_min_ = −0.35 e Å^−^^3^

### Fractional atomic coordinates and isotropic or equivalent isotropic displacement parameters (Å^2^)

**Table:**

	*x*	*y*	*z*	*U*_iso_*/*U*_eq_	
Cl1	1.04341 (9)	0.83154 (3)	−0.08650 (2)	0.05829 (14)	
O1	0.71978 (19)	0.48481 (8)	0.09291 (5)	0.0498 (3)	
O2	1.04495 (18)	0.43162 (7)	0.20029 (5)	0.0401 (2)	
O3	0.8225 (2)	0.53669 (7)	0.24573 (6)	0.0570 (3)	
C1	0.9224 (2)	0.52533 (9)	0.10107 (6)	0.0340 (3)	
C2	1.1344 (2)	0.50163 (11)	0.16112 (7)	0.0408 (3)	
H2A	1.2901	0.4813	0.1440	0.049*	
H2B	1.1806	0.5554	0.1903	0.049*	
C3	0.8633 (3)	0.45846 (9)	0.23619 (7)	0.0370 (3)	
C11	0.9666 (2)	0.59907 (9)	0.05398 (6)	0.0313 (3)	
C12	1.1866 (2)	0.65299 (10)	0.06517 (7)	0.0391 (3)	
H12	1.3210	0.6407	0.1028	0.047*	
C13	1.2108 (3)	0.72426 (10)	0.02184 (8)	0.0430 (3)	
H13	1.3601	0.7614	0.0298	0.052*	
C14	1.0158 (3)	0.74075 (9)	−0.03304 (7)	0.0384 (3)	
C15	0.7980 (3)	0.68741 (10)	−0.04616 (7)	0.0421 (3)	
H15	0.6668	0.6989	−0.0847	0.050*	
C16	0.7743 (2)	0.61712 (9)	−0.00232 (7)	0.0381 (3)	
H16	0.6243	0.5804	−0.0107	0.046*	
C20	0.7172 (2)	0.38165 (8)	0.25836 (6)	0.0336 (3)	
C21	0.7052 (3)	0.30251 (10)	0.22178 (7)	0.0424 (3)	
H21	0.8074	0.2956	0.1873	0.051*	
C22	0.5434 (3)	0.23170 (10)	0.23488 (8)	0.0523 (4)	
H22	0.5388	0.1771	0.2098	0.063*	
C23	0.3936 (3)	0.24148 (10)	0.28341 (8)	0.0505 (4)	
H23	0.2801	0.1942	0.2906	0.061*	
C24	0.4034 (3)	0.32039 (10)	0.32326 (7)	0.0412 (3)	
C25	0.2497 (3)	0.33038 (13)	0.37451 (9)	0.0546 (4)	
H25	0.1335	0.2836	0.3811	0.066*	
C26	0.2647 (3)	0.40503 (14)	0.41426 (9)	0.0597 (5)	
H26	0.1594	0.4106	0.4482	0.072*	
C27	0.4364 (3)	0.47393 (12)	0.40503 (8)	0.0543 (4)	
H27	0.4505	0.5254	0.4339	0.065*	
C28	0.5840 (3)	0.46848 (10)	0.35528 (7)	0.0425 (3)	
H28	0.6973	0.5166	0.3496	0.051*	
C29	0.5706 (2)	0.39209 (9)	0.31191 (7)	0.0340 (3)	

### Atomic displacement parameters (Å^2^)

**Table:**

	*U*^11^	*U*^22^	*U*^33^	*U*^12^	*U*^13^	*U*^23^
Cl1	0.0760 (3)	0.0426 (2)	0.0591 (3)	−0.00666 (18)	0.0197 (2)	0.01078 (17)
O1	0.0353 (5)	0.0632 (7)	0.0466 (6)	−0.0177 (5)	−0.0047 (4)	0.0150 (5)
O2	0.0345 (5)	0.0452 (5)	0.0400 (5)	0.0055 (4)	0.0053 (4)	0.0087 (4)
O3	0.0793 (8)	0.0324 (5)	0.0669 (7)	−0.0031 (5)	0.0335 (6)	0.0017 (5)
C1	0.0268 (6)	0.0416 (7)	0.0324 (6)	−0.0030 (5)	0.0025 (5)	−0.0002 (5)
C2	0.0301 (6)	0.0530 (8)	0.0378 (7)	−0.0022 (6)	0.0024 (5)	0.0075 (6)
C3	0.0379 (7)	0.0374 (7)	0.0340 (6)	0.0006 (5)	0.0024 (5)	0.0033 (5)
C11	0.0265 (6)	0.0362 (6)	0.0313 (6)	−0.0016 (5)	0.0051 (4)	−0.0032 (5)
C12	0.0288 (6)	0.0494 (8)	0.0376 (7)	−0.0067 (5)	0.0018 (5)	−0.0014 (6)
C13	0.0369 (7)	0.0465 (8)	0.0464 (8)	−0.0134 (6)	0.0094 (6)	−0.0025 (6)
C14	0.0457 (7)	0.0334 (6)	0.0387 (7)	−0.0021 (6)	0.0145 (6)	−0.0007 (5)
C15	0.0402 (7)	0.0453 (8)	0.0377 (7)	−0.0023 (6)	−0.0010 (5)	0.0040 (6)
C16	0.0311 (6)	0.0412 (7)	0.0395 (7)	−0.0071 (5)	0.0000 (5)	0.0010 (5)
C20	0.0337 (6)	0.0314 (6)	0.0329 (6)	0.0026 (5)	−0.0020 (5)	0.0048 (5)
C21	0.0510 (8)	0.0368 (7)	0.0358 (7)	0.0036 (6)	−0.0021 (6)	0.0001 (5)
C22	0.0688 (10)	0.0337 (7)	0.0469 (8)	−0.0047 (7)	−0.0100 (7)	−0.0002 (6)
C23	0.0525 (9)	0.0401 (8)	0.0520 (9)	−0.0110 (7)	−0.0095 (7)	0.0150 (7)
C24	0.0335 (7)	0.0443 (7)	0.0416 (7)	0.0013 (6)	−0.0046 (5)	0.0173 (6)
C25	0.0371 (8)	0.0688 (11)	0.0573 (10)	0.0028 (7)	0.0065 (7)	0.0309 (8)
C26	0.0513 (9)	0.0785 (12)	0.0530 (9)	0.0224 (9)	0.0195 (8)	0.0224 (9)
C27	0.0618 (10)	0.0570 (10)	0.0457 (8)	0.0220 (8)	0.0138 (7)	0.0056 (7)
C28	0.0462 (8)	0.0396 (7)	0.0415 (7)	0.0073 (6)	0.0074 (6)	0.0031 (6)
C29	0.0305 (6)	0.0336 (6)	0.0349 (6)	0.0048 (5)	−0.0022 (5)	0.0078 (5)

### Geometric parameters (Å, º)

**Table:**

Cl1—C14	1.7375 (14)	C16—H16	0.9500
O1—C1	1.2110 (15)	C20—C21	1.3765 (19)
O2—C3	1.3523 (17)	C20—C29	1.4305 (19)
O2—C2	1.4279 (16)	C21—C22	1.407 (2)
O3—C3	1.2023 (17)	C21—H21	0.9500
C1—C11	1.4847 (18)	C22—C23	1.358 (2)
C1—C2	1.5206 (18)	C22—H22	0.9500
C2—H2A	0.9900	C23—C24	1.409 (2)
C2—H2B	0.9900	C23—H23	0.9500
C3—C20	1.4862 (18)	C24—C25	1.418 (2)
C11—C16	1.3926 (18)	C24—C29	1.4255 (19)
C11—C12	1.3933 (17)	C25—C26	1.354 (3)
C12—C13	1.384 (2)	C25—H25	0.9500
C12—H12	0.9500	C26—C27	1.400 (3)
C13—C14	1.378 (2)	C26—H26	0.9500
C13—H13	0.9500	C27—C28	1.365 (2)
C14—C15	1.380 (2)	C27—H27	0.9500
C15—C16	1.379 (2)	C28—C29	1.4178 (19)
C15—H15	0.9500	C28—H28	0.9500
			
C3—O2—C2	114.00 (11)	C21—C20—C29	120.29 (12)
O1—C1—C11	121.12 (11)	C21—C20—C3	118.42 (13)
O1—C1—C2	119.71 (12)	C29—C20—C3	120.99 (12)
C11—C1—C2	119.16 (11)	C20—C21—C22	120.81 (15)
O2—C2—C1	109.09 (10)	C20—C21—H21	119.6
O2—C2—H2A	109.9	C22—C21—H21	119.6
C1—C2—H2A	109.9	C23—C22—C21	120.00 (14)
O2—C2—H2B	109.9	C23—C22—H22	120.0
C1—C2—H2B	109.9	C21—C22—H22	120.0
H2A—C2—H2B	108.3	C22—C23—C24	121.30 (14)
O3—C3—O2	122.10 (13)	C22—C23—H23	119.3
O3—C3—C20	125.28 (13)	C24—C23—H23	119.3
O2—C3—C20	112.55 (11)	C23—C24—C25	121.45 (15)
C16—C11—C12	118.80 (12)	C23—C24—C29	119.55 (14)
C16—C11—C1	118.20 (11)	C25—C24—C29	119.00 (15)
C12—C11—C1	122.94 (11)	C26—C25—C24	121.49 (15)
C13—C12—C11	120.50 (12)	C26—C25—H25	119.3
C13—C12—H12	119.7	C24—C25—H25	119.3
C11—C12—H12	119.7	C25—C26—C27	119.55 (15)
C14—C13—C12	119.20 (12)	C25—C26—H26	120.2
C14—C13—H13	120.4	C27—C26—H26	120.2
C12—C13—H13	120.4	C28—C27—C26	121.18 (17)
C13—C14—C15	121.57 (13)	C28—C27—H27	119.4
C13—C14—Cl1	119.20 (11)	C26—C27—H27	119.4
C15—C14—Cl1	119.23 (11)	C27—C28—C29	120.92 (15)
C16—C15—C14	118.84 (13)	C27—C28—H28	119.5
C16—C15—H15	120.6	C29—C28—H28	119.5
C14—C15—H15	120.6	C28—C29—C24	117.78 (13)
C15—C16—C11	121.07 (12)	C28—C29—C20	124.27 (12)
C15—C16—H16	119.5	C24—C29—C20	117.94 (12)
C11—C16—H16	119.5		
			
C3—O2—C2—C1	−71.19 (14)	O2—C3—C20—C29	162.30 (11)
O1—C1—C2—O2	−1.16 (19)	C29—C20—C21—C22	1.9 (2)
C11—C1—C2—O2	177.87 (11)	C3—C20—C21—C22	−171.84 (13)
C2—O2—C3—O3	−14.99 (19)	C20—C21—C22—C23	1.0 (2)
C2—O2—C3—C20	162.17 (10)	C21—C22—C23—C24	−2.4 (2)
O1—C1—C11—C16	−3.7 (2)	C22—C23—C24—C25	−179.23 (14)
C2—C1—C11—C16	177.32 (12)	C22—C23—C24—C29	0.9 (2)
O1—C1—C11—C12	173.71 (14)	C23—C24—C25—C26	177.95 (14)
C2—C1—C11—C12	−5.3 (2)	C29—C24—C25—C26	−2.2 (2)
C16—C11—C12—C13	1.3 (2)	C24—C25—C26—C27	−0.3 (2)
C1—C11—C12—C13	−176.09 (13)	C25—C26—C27—C28	1.9 (2)
C11—C12—C13—C14	−0.7 (2)	C26—C27—C28—C29	−0.9 (2)
C12—C13—C14—C15	−0.6 (2)	C27—C28—C29—C24	−1.65 (19)
C12—C13—C14—Cl1	178.78 (11)	C27—C28—C29—C20	179.49 (13)
C13—C14—C15—C16	1.2 (2)	C23—C24—C29—C28	−177.01 (12)
Cl1—C14—C15—C16	−178.12 (11)	C25—C24—C29—C28	3.14 (18)
C14—C15—C16—C11	−0.6 (2)	C23—C24—C29—C20	1.92 (18)
C12—C11—C16—C15	−0.6 (2)	C25—C24—C29—C20	−177.93 (11)
C1—C11—C16—C15	176.89 (13)	C21—C20—C29—C28	175.54 (12)
O3—C3—C20—C21	153.08 (15)	C3—C20—C29—C28	−10.86 (19)
O2—C3—C20—C21	−23.97 (16)	C21—C20—C29—C24	−3.32 (17)
O3—C3—C20—C29	−20.6 (2)	C3—C20—C29—C24	170.29 (11)

### Hydrogen-bond geometry (Å, º)

**Table:**

*D*—H···*A*	*D*—H	H···*A*	*D*···*A*	*D*—H···*A*
C16—H16···O1^i^	0.95	2.41	3.2583 (16)	148
C23—H23···O3^ii^	0.95	2.48	3.2618 (19)	140

Symmetry codes: (i) −*x*+1, −*y*+1, −*z*; (ii) −*x*+1, *y*−1/2, −*z*+1/2.
